# Assessing the reliability of DP and Fermi estimates in single and dual bolus cardiac MR perfusion imaging

**DOI:** 10.1186/1532-429X-16-S1-P347

**Published:** 2014-01-16

**Authors:** Giorgos Papanastasiou, Michelle C Williams, Shirjel Alam, Mark Dweck, Saeed Mirsadraee, Calum D Gray, Martin Connell, Thomas J MacGillivray, David Newby, Scott Semple

**Affiliations:** 1 Clinical Research Imaging Center, University of Edinburgh, Edinburgh, Lothian, UK; 2 Centre for Cardiovascular Science, University of Edinburgh, Edinburgh, Lothian, UK

## Background

MR dual bolus (DB) perfusion imaging has been effectively shown to eliminate signal saturation of the arterial input function (AIF) and allow more reliable quantification of myocardial (Myo) blood flow (MBF). Single bolus (SB) protocols are prone to AIF signal saturation but have been widely used in clinical studies. The Fermi model has been well established for MBF quantification. The distributed parameter (DP) model has been implemented in a recent study to measure additional physiological parameters (PP) such as intravascular (vb) and extravascular-extracellular space (ve) of the coronary arteries (CAs). Our study aims to a) validate absolute values, correlations and differences between SB versus DB estimates and b) assess which model more reliably fits SB data.

## Methods

After informed consent, 8 healthy volunteers underwent adenosine stress-rest MR myocardial perfusion imaging (3T Verio, Siemens AG, Healthcare Sector). A dilute (0.006 mmol/kg) Gd-based contrast agent (Gadovist, Bayer Healthcare) was injected to allow extraction of the AIF for the DB analysis followed by a standard (0.03 mmol/kg) Gd dose for the extraction of Myo signal intensity curves (Ishida M et al. 2011). Images were acquired in mid-diastole. SB AIF data were generated by extracting the AIF from the standard Gd dose component of the DB data. Signal intensity AIF and Myo curves were converted to Gd concentration curves (Biglands J et al. 2011). The Fermi model (Jerosch-Herold M et al. 1998) was used to quantify MBF as a reference standard against DP-MBF (Broadbent et al. 2013). DP and Fermi values were examined both in SB and DB data. Bland Altman plots and paired t-test (p values < 0.01 were considered significant) were used for data analysis.

## Results

**Figure 1:**
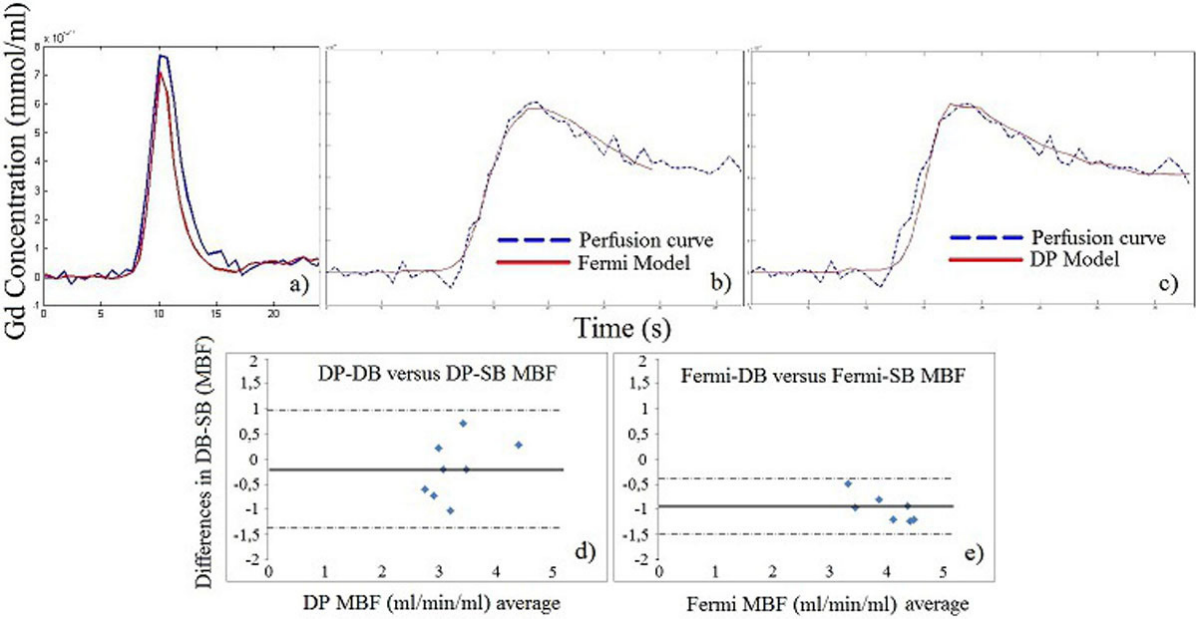
**Scaled DB AIF (blue) versus SB AIF (red) under adenosine stress**. In SB AIF, the first-pass of Gd (first pass: from the beginning of contrast enhancement until just before re-circulation occurs) in (1a) is prone to saturation. Saturation results in lower Gd concentrations (SB is lower than DB AIF at peak and up to the end of first pass). b) Fermi model is fitted to the first pass and c) DP model fitted in all data points. Bland Altman plots assessing bias between DB and SB values (in 8 healthy volunteers) in d) DP model (bias value = 0.19, 95% confidence intervals [-1.32, 0.95]) and e) Fermi model (bias value= -0.93, 95% confidence intervals [-1.47, -0.4]). No significant change was observed in DP whilst Fermi-SB MBF values were significantly higher than Fermi-DB.

The most significant difference between SB and DB parameter estimates was observed in the Fermi model during stress (Table 1 p < 0.01). Fermi values were influenced by saturation at the AIF peak because the Fermi model fits only to the first pass Myo curves (Figure 1a). There was no significant difference between DP-SB and DP-DB values: MBF (p = 0.38, p = 0.89), ve (p = 0.02, p = 0.04) and permeability surface area product (PS) (p = 0.11, p = 0.02), at stress and rest respectively. Our estimates of PS are lower than those reported by Broadbent et al's study which used mid-systolic data. This suggests that DP may be capable of detecting lower Gd permeability from vb into ve which is associated with relaxation of the subendocardial CAs during mid-diastole. In our study, DP was fitted for the first time in mid-diastole, which is the preferable method for perfusion acquisition.

**Table 1:** Parameter comparisons are shown in SB and DB data both at stress (S) and rest (R).

Modeling values/ Method	DP-DB	DP-SB	Fermi-DB	Fermi-SB
MBF-S (ml/min/ml)	3.20 (0.97)	3.40 (0.74)	3.60 (0.79)	4.50 (0.80)

MBF-R (ml/min/ml)	1.23 (0.37)	1.45 (0.40)	1.47 (0.50)	1.56 (0.45)

PS-S (ml/min/ml)	0.98 (0.32)	1.09 (0.21)		

PS-R (ml/min/ml)	0.56(0.15)	0.62 (0.14)		

E-S	0.45(0.04)	0.45(0.03)		

E-R	0.56(0.04)	0.54(0.03)		

vb-S	0.08(0.02)	0.09(0.02)		

vb-R	0.04(0.01)	0.04 (0.01)		

ve-S	0.17(0.05)	0.20(0.04)		

ve-R	0.17(0.06)	0.20(0.05)		

The extraction fraction (E) and vb did not change in SB versus DB analysis: E-S (p = 0.90), E-R (p = 0.10), vb-S (p = 0.09), vb-R (p = 0.68). Mean values ( ± SD) are shown.

## Conclusions

DP-MBF and PP values were not significantly affected when SB AIF was used which suggests that DP model might more reliably model SB data than Fermi. This should be further investigated in larger populations. PS was low compared to previous mid-systole analysis, suggesting that DP may show potential in detecting decreased permeability of the CAs in mid-diastole.

## Funding

This work was made possible through funding and continued support from the British Heart Foundation.

